# Supplementary material for: An Optimization of Oregano, Thyme, and Lemongrass Essential Oil Blend to Simultaneous Inactivation of Relevant Foodborne Pathogens by Simplex–Centroid Mixture Design
Source: Antibiotics (Basel). 2022 Nov 8;11(11):1572. doi: 10.3390/antibiotics11111572 (PMC9686886; doi:10.3390/antibiotics11111572)
Supplement: Supplementary file 1 [file antibiotics-11-01572-s001.zip › antibiotics-1998752-supplementary.pdf]

Supplementary Table S1. Composition of oregano (ORE; *Origanum vulgare*), thyme (THY; *Thymus vulgaris*), and lemongrass (LG; *Cymbopogon citratus*) essential oils through gas chromatography coupled to mass spectrometry (GC-MS) and gas chromatography coupled to flame ionization detector (CG-FID).

Cymbopogon citratus					*Origanum vulgare*					*Thymus vulgaris*				
Peak	¥RI exp	€RI lit	Identification	Area %**	Peak	RI exp	RI lit	Identification	Area %**	Peak	RI exp	RI lit	Identification	Area %**
1	920	921	tricyclene	tr	1	920	921	tricyclene	0.1	1	930	932	alpha-pinene	3.0
2	930	932	alpha-pinene	0.1	2	924	924	alpha-tujene	0.0	2	944	946	camphene	2.2
3	945	946	camphene	0.3	3	930	932	alpha-pinene	1.2	3	988	988	mircene	1.3
4	987	981	6-methyl-5-hepten-2-one	0.4	4	945	946	camphene	0.4	4	1021	1022	p-cymene	21.7
5	990	988	dehydro-1,8-cineole	0.1	5	973	974	beta-pinene	0.3	5	1025	1024	limonene	3.4
6	1025	1024	limonene	0.9	6	977	974	1-octen-3-ol	0.1	6	1028	1026	1,8-cineole	2.5
7	1034	1032	(Z)-beta-ocymene	0.4	7	980	-	n.i.	0.1	7	1099	1098	linalool	6.0
8	1044	1044	(E)-beta-ocymene	0.2	8	988	988	myrcene	1.6	8	1162	1165	borneol	3.4
9	1070	-	4-nonanone	0.3	9	1003	1002	alpha-phelandrene	0.2	9	1291	1289	thymol	31.2
10	1099	1098	linalool	0.6	10	1008	1008	delta-3-carene	0.1	10	1301	1298	carvacrol	25.5
11	1103	-	n.i.	0.1	11	1014	1014	alpha-terpinene	1.0					
12	1142	-	n.i.	0.6	12	1019	1020	m-cymene	0.1					
13	1146	-	n.i.	0.2	13	1022	1022	p-cymene	10.4					
14	1150	1148	citronellal	1.3	14	1025	1024	limonene	0.6					
15	1162	-	n.i.	1.8	15	1028	1026	1,8-cineole	0.5					
16	1180	-	n.i.	2.6	16	1054	1054	gamma-terpinene	4.8					
17	1227	1223	citronellol	1.1	17	1099	1098	linalool	2.2					
18	1237	1235	neral	33.7	18	1162	1165	borneol	0.2					
19	1253	1249	geraniol	4.5	19	1174	1174	terpinen-4-ol	0.4					
20	1266	1264	geranial	45.5	20	1292	1289	thymol	0.4					
21	1351	1350	citronellyl acetate	0.2	21	1300	1298	carvacrol	70.3					
22	1381	1379	geranyl acetate	1.5	22	1412	1417	(E)-beta-caryophyllene	4.7					

23	1386	1389	beta-elemene	0.4	23	1445	1452	alpha-humulene	0.1	
24	1411	1417	(E)-beta-caryophyllene	0.9	24	1575	1582	caryophyllene oxide	0.3	
25	1445	1452	alpha-humulene	0.1						
26	1473	1480	germacrene D	0.2						
27	1506	1513	gamma-cadinene	0.4						
28	1516	1522	delta-cadinene	0.3						
29	1543	1548	elemol	0.9						
30	1575	1572	caryophyllene oxide	0.5						
Total:				100.0					100.0	100
Total identified:				94.8					99.9	100
Monoterpenes:				1.9					20.7	31.5
Oxygenated monoterpenoids:				93.7					74.0	68.5
Sesquiterpenes:				2.3					4.8	-
Oxygenated sesquiterpenoids:				1.5					0.3	-
Others:				0.6					0.1	-

n.i.: Not identified
tr: trace (<0.1%)
¥RI exp: Experimental Retention Index;
€RI lit: Literature Retention Index (Adams);
** Normalized and corrected percentage area with response factors.
